# Changing Trends of Respiratory Viruses in Hospitalized Children During and After the COVID-19 Emergency Phase in Yongin, South Korea (2020–22 vs. 2023–24)

**DOI:** 10.3390/v18010130

**Published:** 2026-01-20

**Authors:** Joon-sik Choi, Eun Gyeong Seol, Ji Hyun Lee, Heejung Kim, Kyung Min Choi, Min Jung Kim

**Affiliations:** 1 Department of Pediatrics, Gangnam Severance Hospital, Yonsei University College of Medicine, Seoul 06273, Republic of Korea; 2 Department of Pediatrics, Yongin Severance Hospital, Yonsei University College of Medicine, Yongin-si 16995, Republic of Korea; 3 Department of Hospital Medicine, Yongin Severance Hospital, Yonsei University College of Medicine, Yongin-si 16995, Republic of Korea; 4 Department of Laboratory Medicine, Yongin Severance Hospital, Yonsei University College of Medicine, Yongin-si 16995, Republic of Korea

**Keywords:** child, COVID-19, immunity, respiratory viruses, public health

## Abstract

The COVID-19 pandemic and subsequent non-pharmaceutical interventions (NPIs) significantly disrupted the epidemiology of pediatric respiratory viruses. This study compared infection patterns among 3658 hospitalized children in South Korea during the pandemic (2020–2022) and the post-emergency phase (2023–2024), following the relaxation of mandatory NPIs. Of 4419 eligible tests, the most frequently detected viruses overall were rhinovirus/enterovirus (HRV/HEV) (27.9%), influenza (14.5%), and respiratory syncytial virus (RSV, 11.9%). The post-emergency phase was marked by a dramatic surge in influenza virus (IFV), which surged dramatically (5.5% → 28.2%), and a more than two-fold increase in adenovirus (ADV) (5.7% → 12.5%) (*p* < 0.001). (*p* < 0.001). Conversely, parainfluenza virus (PIV) detection rates declined significantly (15.4% → 11.3%, *p* < 0.001). Demographically, post-emergency phase patients were significantly older (mean 4.9 vs. 3.5 years) and experienced a shorter hospital stays (3.2 vs. 4.3 days) (*p* < 0.001). Crucially, age-specific susceptibility shifts were evident. IFV rebounded across all pediatric ages but spiked severely in school-aged children and adolescents, while HRV/HEV demonstrated a clear proportional shift towards older age groups. These results demonstrate a substantial reconfiguration of the pediatric respiratory landscape, necessitating age-stratified surveillance and flexible public health strategies to mitigate the future infectious disease burden.

## 1. Introduction

Acute respiratory infections (ARIs) are the most common type of childhood illnesses, occurring six to eight times a year and representing a leading cause of pediatric hospitalizations [1,2]. ARIs, particularly in children under two years of age, can impose significant emotional, logistical, and financial burdens on families and caregivers [3]. Most pediatric ARIs are viral and caused by well-known agents such as human rhinovirus (HRV), respiratory syncytial virus (RSV), parainfluenza viruses (PIV), human metapneumovirus (MPV), adenovirus (ADV), bocavirus, influenza viruses (IFV), and seasonal coronaviruses (CoV) [1]. Each virus tends to exhibit distinct age-related susceptibility patterns, well-known symptom profiles, and predictable seasonal peaks, which aid in guiding clinical decision-making and public health strategies [4]. However, following the global emergence of a new coronavirus (Severe acute respiratory syndrome coronavirus 2 [SARS-CoV-2]) in late 2019, the coronavirus disease 2019 (COVID-19) pandemic has significantly altered the landscape of respiratory viruses and continues to shape respiratory virus epidemiology [5,6].

Non-pharmaceutical interventions (NPIs) implemented early in the pandemic, including social distancing, mask-wearing, and thorough hand hygiene, led to an unprecedented decline in the global spread of common respiratory viruses [5,6,7,8]. These observations have been discussed under the “immunity debt” hypothesis; however, the concept remains debated and likely reflects multiple interacting mechanisms, including delayed exposure and a transient susceptibility gap at the population level, ecological interactions between viruses, changes in healthcare-seeking/testing behavior, and possible immune modulation after SARS-CoV-2 infection [9,10,11]. All of these changes have led to higher infection rates, shifts in co-infection patterns, and increased hospitalizations. These changes create new challenges for healthcare providers and add extra pressure to healthcare systems already strained by the pandemic [12,13].

Yongin Severance Hospital, which opened in March 2020 at the start of the COVID-19 outbreak in Korea, could offer a unique perspective on the evolving dynamics during and after the pandemic. Leveraging this unique time frame and institutional data, this study analyzed changes in the prevalence of common respiratory viruses among hospitalized children with acute respiratory symptoms during and after the COVID-19 outbreak. We primarily focused on comparing the COVID-19 pandemic phase (2020–2022) to the post-emergency phase (2023–2024), aiming to understand how the pandemic has transformed the landscape of respiratory virus infections in children and to explore future public health implications.

## 2. Materials and Methods

### 2.1. Study Setting and Population

This study included data collected from March 2020 to December 2024. The COVID-19 emergency phase was defined as March 2020 to December 2022, whereas the post-emergency phase began in January 2023 and continued until the end of the study. The post-emergency phase followed the relaxation of NPIs, especially the lifting of the indoor mask mandate for most public spaces, including schools, in South Korea. This study was approved by the Institutional Review Board of Yongin Severance Hospital (Yongin, South Korea) (protocol no. 9-2023-0253). All protocols and methods described in this study were conducted in accordance with relevant guidelines and regulations. The requirement for informed consent was waived owing to the study’s retrospective nature and the use of anonymized patient data.

The study population included patients aged from one month to under 18 years who had been admitted to or visited the emergency department of Yongin Severance Hospital for acute respiratory symptoms. Patients were grouped into the following age categories: infants (younger than 1 year), toddlers (1 year or older but younger than 4 years), preschool-aged children (4 years or older but younger than 8 years), school-aged children (8 years or older but younger than 13 years), and adolescents (13 years or older but younger than 18 years). Demographic and clinical data, including diagnoses and laboratory test results, were collected through a retrospective review of medical charts.

### 2.2. Respiratory Pathogen Detection

Respiratory viruses were identified using two available methods, both of which employ the polymerase chain reaction (PCR) technique. Since the hospital opened in March 2020, multiplex real-time PCR (RT-PCR) [Seegene Allplex Respiratory Panel 1, 2, 3 (Seegene Inc., Seoul, Republic of Korea)] has been used to detect respiratory viruses. However, bioMérieux BioFire FilmArray^®^ RP (BioFire Diagnostics, Salt Lake City, UT, USA) has become more widely used since March 2022 due to its advantages over other techniques, including faster test results and a broader detection range of pathogens such as SARS-CoV-2, Middle East respiratory syndrome coronavirus (MERS-CoV), Mycoplasma pneumoniae, and Bordetella pertussis. Given the clear strengths and weaknesses of each testing method, clinicians can choose one based on specific needs (Table 1). Although additional microbiologic tests, such as bacterial infections, were performed at clinicians’ discretion, only multiplex RT-PCR viral results were used for the primary analyses in this study. For influenza cases, positive rapid antigen test results were also recorded. SARS-CoV-2 detection was possible with both FilmArray^®^ and single-target RT-PCR tests. If any targeted virus was identified in the specimen, the patient tested positive for that specific virus. When two or more viruses were detected in the same clinical sample, each was counted separately.

### 2.3. Statistical Analysis

Categorical variables were expressed as numbers and percentages. Baseline characteristics of the patients were analyzed using the Mann–Whitney U test or Fisher’s exact test, as appropriate. Normality of the distribution was assessed with the Kolmogorov–Smirnov test. Numerical variables are presented as means and standard deviations (SDs). For variables with non-normal distributions, we used the median and interquartile range (IQR). Statistical significance was defined as *p* < 0.05. All analyses were performed with IBM SPSS Statistics version 27.0 (IBM Corp., Armonk, NY, USA), and graphs were created in Microsoft Excel (Microsoft Corp., Redmond, WA, USA) and ChatGPT-4o (OpenAI, San Francisco, CA, USA). All outputs were verified by the authors.

## 3. Results

### 3.1. Overall Detection and Shifts in Seasonal Patterns of Respiratory Viruses

During the study period, a total of 5173 individuals were tested. After excluding duplicate cases and bacterial findings, data from 4419 tests, including 3658 patients, were included in the final analysis (1894 multiplex RT-PCR tests, 1513 Film Array tests, 486 single RT-PCR tests, and 526 antigen tests).

**Figure 1:**
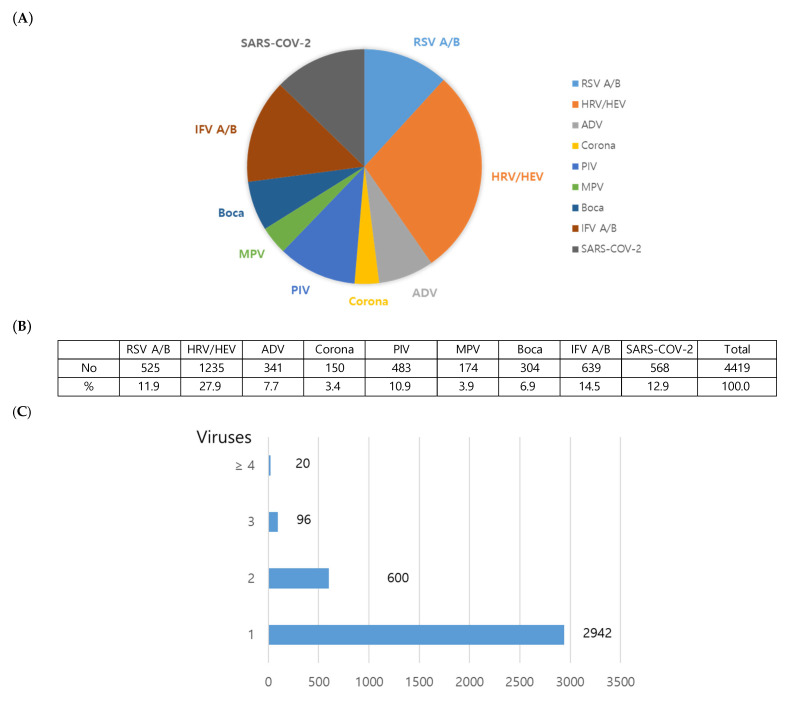
Distribution of detected viruses. (**A**,**B**) Percentage of detected viruses in the study period. (**C**) Counts of cases with co-infections in subjects.

The distribution of the detected viruses is presented in Figure 1. The most frequently detected respiratory virus throughout the study was the HRV group, which included HRV and HEV (Figure 1A,B). Most patients (2942 out of 3658; 80.4%) were infected with a single virus (Figure 1C). The HRV group was the most prevalent virus among cases with multiple infectious agents. The most common dual co-infection was HRV and RSV, observed in 122 patients (17.0% of co-infections).

**Figure 2:**
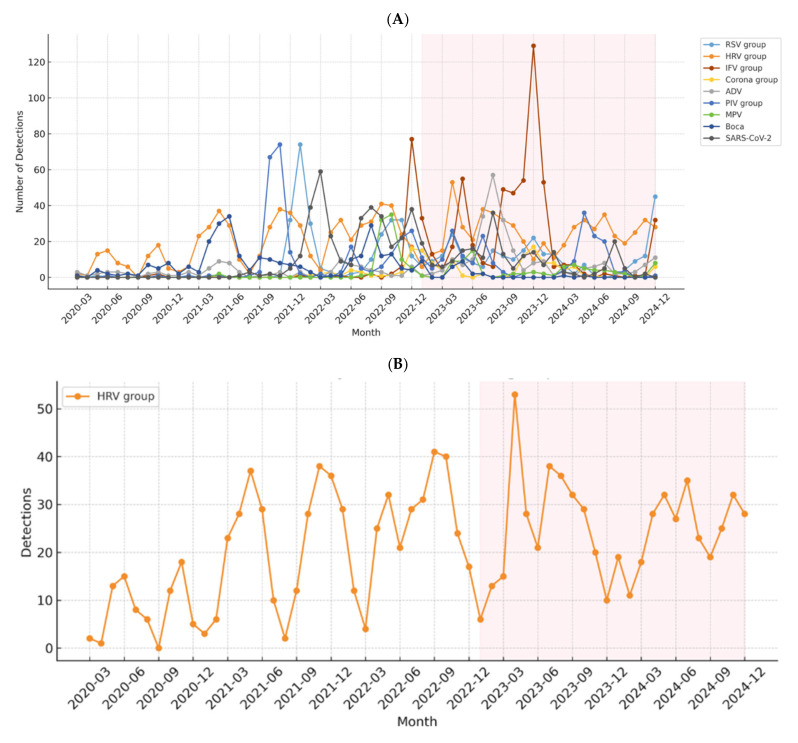

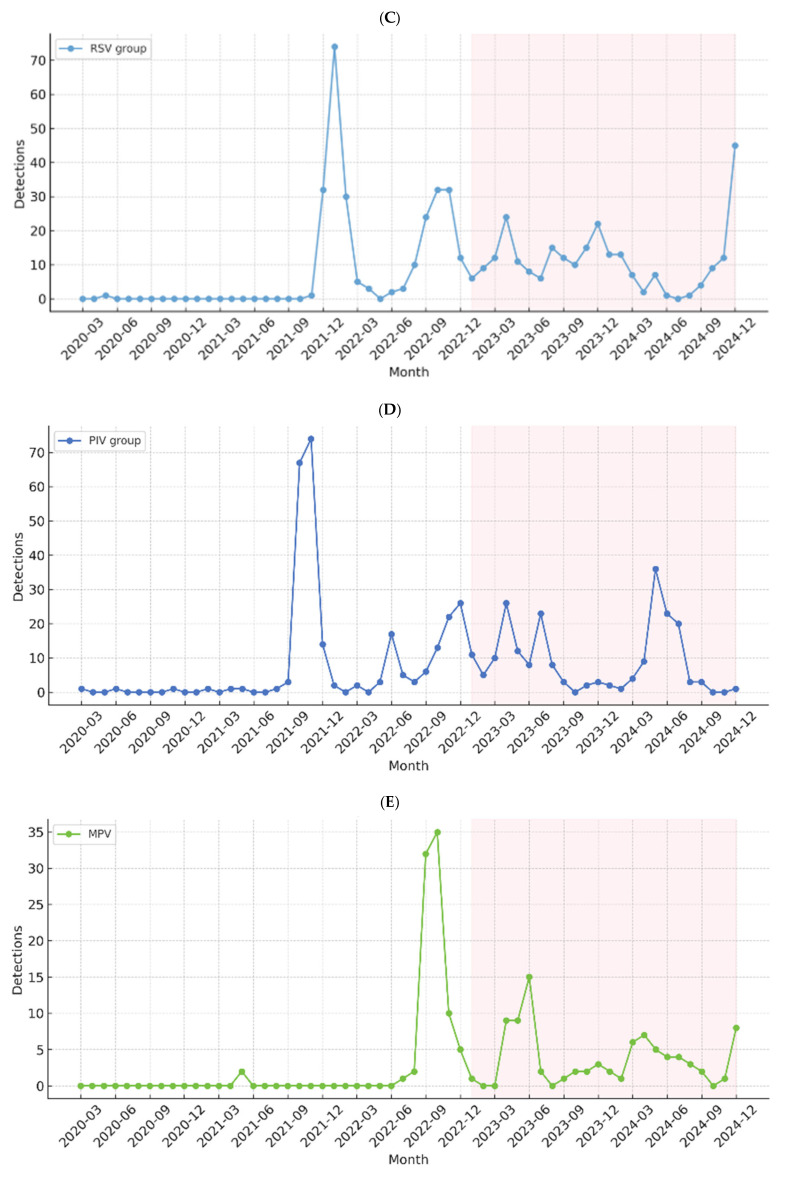

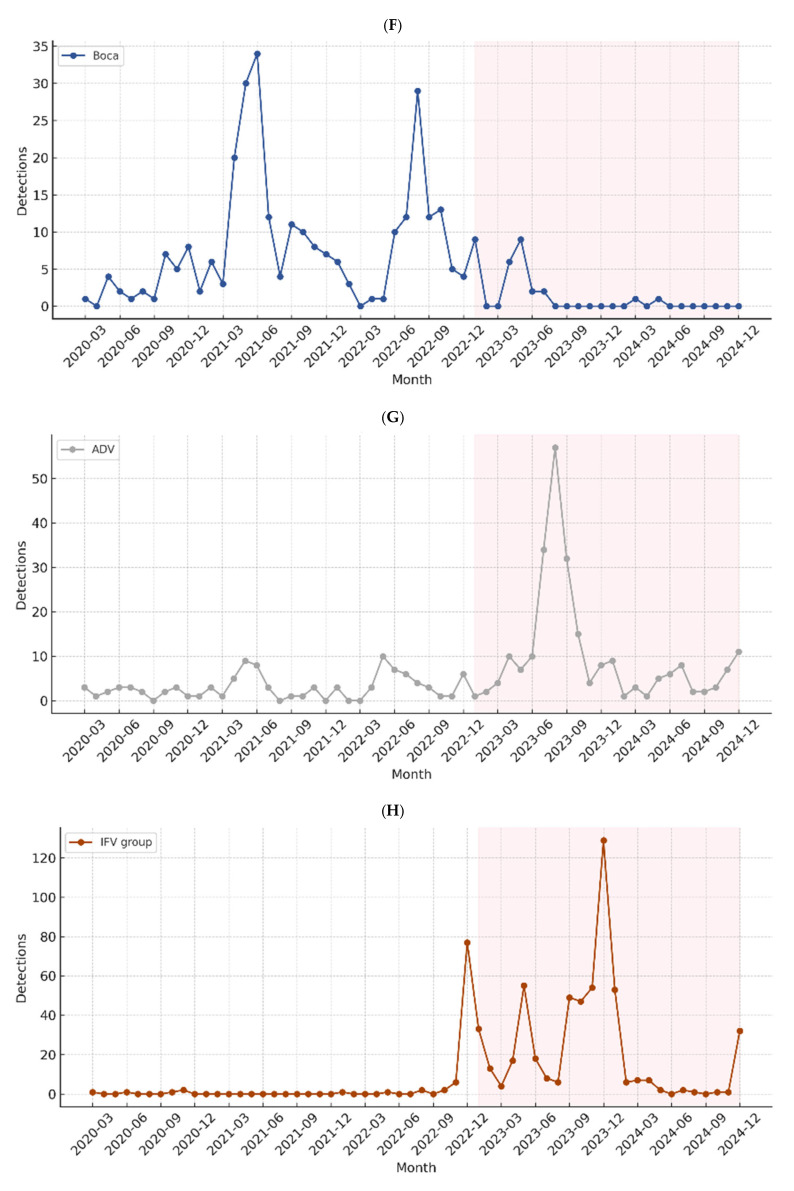

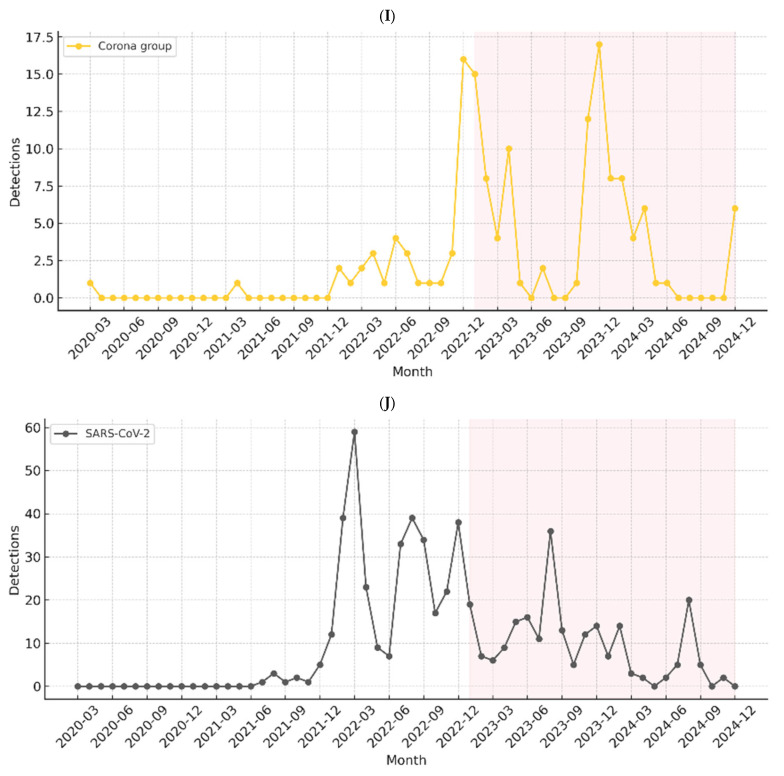
Detection of respiratory viruses during and after the COVID-19 emergency phase. (**A**) Patterns in detection of respiratory viruses during and after the COVID-19 emergency phase (**B**–**J**) Changes in detection of individual respiratory viruses during and after the COVID-19 emergency phase (**B**) HRV/HEV, (**C**) RSV A and B, (**D**) PIV 1–4, (**E**) MPV, (**F**) Bocavirus, (**G**) ADV, (**H**) IFV A and B, (**I**) Coronaviruses; (**J**) SARS-CoV-2.

The patterns of overall and individual respiratory virus infections during the study period are presented in Figure 2. There was a significant decline in the detection of almost all respiratory viruses at the start of the COVID-19 emergency, followed by a noticeable rebound throughout the emergency phase and during the post-emergency phase (Figure 2A). HRV/HEV was relatively less impacted by the COVID-19 emergency and remained prevalent throughout the study period (Figure 2B). RSV was virtually absent in early 2020 but re-emerged with an outbreak in late 2021, followed by another surge in the summer of 2022. By the 2023–2024 season, RSV infections had largely returned to their pre-pandemic seasonal pattern (Figure 2C). The PIV group was heavily impacted, initially exhibiting almost no activity, followed by an unusual surge in late 2021 (peaking above 70 detections). Overall, PIV detection rates decreased slightly from 15.4% to 11.3% after the COVID-19 emergency, but its activity exhibited recurring spring-summer seasonality in 2023 and 2024, unlike the pre-pandemic pattern (Figure 2D).

In contrast, MPV displayed relatively stable detection rates across both periods (Figure 2E). Notably, IFV A/B was almost undetected during the COVID-19 emergency but surged sharply afterward (Figure 2F), while ADV and endemic CoV also demonstrated substantial post-emergency increases (Figure 2G,I).

Bocavirus detection was low due to a methodological change that excluded it from the FilmArray test. SARS-CoV-2 was rarely detected in this study, as most cases were tested at external screening clinics outside the hospital [14] (Figure 1A,B). These findings are also reflected in the patterns of individual viruses (Figure 2I,J).

### 3.2. Demographic Characteristics and Respiratory Virus Detection During and After the COVID-19 Emergency Phase

Demographic characteristics of the two groups of hospitalized children (*n* = 3658) in Table 2, including 1724 of whom were treated during the COVID-19 emergency and 1934 of whom were treated in the post-emergency phase. The two groups were similar in sex distribution (*p* = 0.795) but differed in age and length of stay (LOS). Post-emergency children were older than those treated during the COVID-19 emergency phase (mean 4.9 ± 5.0 vs. 3.5 ± 4.1 years; *p* < 0.001) and experienced a shorter LOS (days) than those during the COVID-19 emergency phase (3.2 ± 2.6 vs. 4.3 ± 2.7 days; *p* < 0.001).

Marked shifts in virus detection patterns are also presented in Table 2. Detection rates for IFV A/B exhibited the most significant increase, rising from 5.5% (94/1742) during the COVID-19 emergency to 28.2% (545/1934) in the post-emergency phase (*p* < 0.001). Similarly, the detection rate for ADV more than doubled, rising from 5.7% to 12.5% (*p* < 0.001). Conversely, detection of the PIV group declined significantly from 15.4% to 11.3% (*p* < 0.001). The HRV/HEV group also exhibited a statistically significant, albeit more modest, decrease from 36.9% to 30.9% (*p* = 0.001). Overall detection rates for RSV A/B (15.1% vs. 13.7%; *p* = 0.217) and MPV (5.0% vs. 4.5%; *p* = 0.484) did not change significantly between the two periods.

### 3.3. Age Distribution in Cases of Respiratory Virus Infections During and After the COVID-19 Pandemic

Table 3 summarizes the distribution of detected respiratory viruses by age group during the COVID-19 emergency (2020–2022) and the post-emergency (2023–2024) phases. Across both phases, HRV/HEV remained the most frequently detected pathogen, although its age distribution shifted. During the COVID-19 emergency, infections were concentrated in infants (38.1%) and toddlers (45.6%), whereas after the pandemic, the proportion of cases increased in older age groups, including pre-schoolers (32.4%) and school-aged children (23.8%). RSV detection rates exhibited a distinct temporal pattern. RSV circulation was markedly reduced during the early COVID-19 emergency phase, and detection rates rebounded as it abated, leading to an overall increase in the proportion of cases among older age groups. Infants and toddlers continued to account for the most significant proportion of RSV cases in the post-emergency phase (26.3% and 20.7%, respectively). PIVs were predominantly identified in younger children in both phases, but their overall detection rates decreased slightly after the COVID-19 emergency. In contrast, ADV exhibited a substantial post-emergency surge, particularly among toddlers (from 8.1% to 18.2%) and pre-schoolers (from 3.6% to 18.5%). The prevalence of MPV remained relatively stable across both phases, with only minor variations among age groups. IFVs demonstrated the most dramatic shift. During the COVID-19 emergency, IFV detections were rare across all age groups, with influenza B virtually absent. After the COVID-19 emergency, the IFV detection rates increased sharply in all pediatric age groups, particularly among school-aged children (54.5%) and adolescents (69.3%).

**Figure 3:**
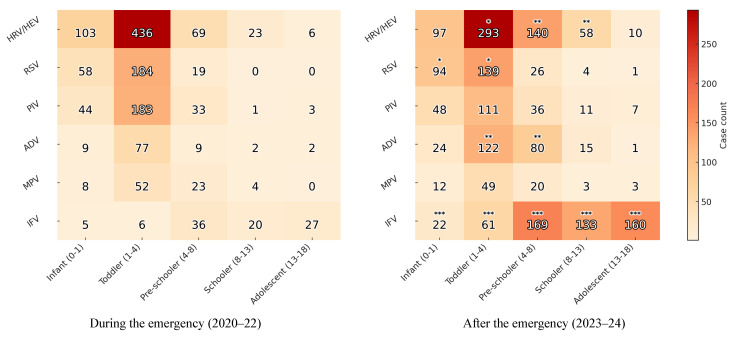
Comparison and significance of age distributions during and after the COVID-19 emergencyphase * *p* < 0.05, ** *p* < 0.01, *** *p* < 0.001. Abbreviations: HRV, Human rhinovirus, HEV, Human enterovirus, RSV, Respiratory syncytial virus, PIV, parainfluenza virus, ADV, adenovirus, MPV, Metapneumovirus, IFV; influenza virus.

**Figure 4:**
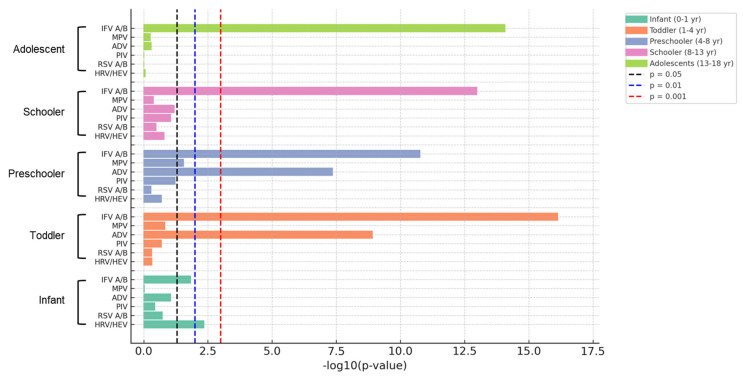
Significant differences in virus detection among different age groups. Statistically significant differences in respiratory virus detection rates among hospitalized children, stratified by age group, during (2020–2022) and after (2023–2024) the COVID-19 emergency phase. Each bar represents an age group–virus combination that showed a statistically significant change in detection frequency between the two periods. The x-axis presents the negative logarithm of the *p*-value (−log_10_(*p*)), providing a clearer visual interpretation of statistical significance. The larger the bar, the more significant the difference. Dashed vertical lines denote conventional thresholds for statistical significance: Gray: *p* = 0.05, Blue: *p* = 0.01, Red: *p* = 0.001. Abbreviations: HRV, Human rhinovirus; HEV, Human enterovirus; RSV, Respiratory syncytial virus; PIV, parainfluenza virus; ADV, adenovirus; MPV, Metapneumovirus; IFV; influenza virus.

These observations are illustrated in Figure 3, which presents significant age–virus combinations with altered detection frequencies after the pandemic. To intuitively present the statistical significance of the change in virus detection between the two periods, the age-specific *p*-values for each virus are expressed on a log scale (Figure 4).

## 4. Discussion

The COVID-19 pandemic and subsequent NPIs have caused notable seasonal and endemic shifts in the epidemiology of common respiratory virus-related illnesses. Yongin Severance Hospital began serving patients at the beginning of the COVID-19 pandemic in Korea. As a general hospital that is easily accessible to the public, it experienced a high volume of visits from both severe cases and patients with mild or moderate respiratory infections. This present study offers insights into the shifts in respiratory virus patterns during the COVID-19 emergency and post-emergency phases, partly influenced by the unique circumstances of the study site. Notable changes included an age shift in HRV/HEV infections toward older children, a resurgence of RSV that affected a broader age range, a significant increase in IFV infections across all ages, and a marked rise in ADV among children aged 1–8 years during the post-emergency phase. These results may indicate that the relaxation of NPIs during the post-emergency phase, changes in contact patterns and delayed exposure may have contributed to shifts in both the circulation patterns and age distribution of common pediatric respiratory viruses. Before the COVID-19 pandemic, pediatric respiratory viruses followed a predictable seasonal pattern worldwide, including winter peaks of RSV and IFV infections, spring and autumn surges of HRV, and sporadic year-round ADV activity [8,15]. The global spread of SARS-CoV-2 in early 2020 and the subsequent implementation of NPIs such as mask mandates, physical distancing, school closures, and travel restrictions fundamentally disrupted those patterns [13]. In 2020, the year in which NPIs were most strictly enforced, surveillance and hospital data from East Asia and other regions showed sharp declines (40–95%) in positivity rates and healthcare utilization for IFV, PIV, ADV, and several other pathogens, along with a 75% drop in direct medical costs [5,7,16]. Environmental factors also played a role. For example, studies from Korea and other countries have demonstrated that improved air quality and reduced mobility during lockdowns were associated with lower viral circulation [17,18], whereas the relaxation of restrictions coincided with viral resurgence [19,20].

Despite any precautionary measures to curb transmission, respiratory viruses never fully disappeared. In many places, they reappeared out of season during the summer–autumn of 2021 as restrictions were relaxed (referred to as off-season resurges) [19,20]. In a group of 41 U.S. children’s hospitals, bronchiolitis admissions decreased by 69% in the 2020–21 season and then increased by 75% in 2022–23, with the seasonal peak shifting to November and the median age at admission rising [16,18]. RSV reemerged outside typical months with a higher median age of infection and longer hospital stays [18,21]. IFVs, especially IFV B that was almost absent during the COVID-19 emergency phase, exhibited a sharp rebound across all pediatric age groups [22,23,24]. ADV infections also increased significantly, particularly among children aged 1–8 years [25]. Immunity gap may also be driven by deferred viral exposure during the NPI era, along with shifts in host susceptibility and changes in community transmission patterns [9]. The altered age distribution of HRV/HEV and RSV may require adjustments in clinical vigilance and prevention strategies, including vaccination timing and prioritization [18,26].

Our findings are consistent with an immunity gap resulting from reduced viral exposure during the NPI era, although the observed patterns are likely multifactorial and may also reflect behavioral changes and ecological interactions among viruses during the post-emergency phase [10,27]. The global implementation of NPIs during the COVID-19 emergency led to a period of reduced viral exposure, inadvertently delaying the development of immunity in susceptible cohorts, which subsequently resulted in off-season resurges and age shifts once restrictions were relaxed worldwide. In our study, HRV/HEV, which maintained a relatively high prevalence even during the strictest NPIs, demonstrated a clear shift in infection proportion toward older children (4 years and older) in the post-emergency phase. This suggests that while infants and toddlers continue to experience infection, school-aged children and adolescents, who were protected by prolonged school closures and mask mandates, experienced deferred primary or secondary exposures once NPIs were lifted. Similarly, ADV exhibited a substantial post-pandemic surge, particularly among toddlers (1–4 years) and preschoolers (4–8 years).

The most dramatic evidence of the immunity gap was the widespread rebound of IFV across all age groups, peaking significantly in school-aged children and adolescents [25]. This rebound followed its near-complete absence in the COVID-19 emergency phase, highlighting the impact of two years of minimal circulation on population immunity. Furthermore, while RSV exhibited atypical seasonality (with abrupt, unusual surges even in the summer) during the COVID-19 emergency phase, its return to the typical strong winter-dominant pattern in 2023–2024 (as presented in Figure 2) suggests a key step toward the normalization of seasonal viral patterns, potentially reflecting the restoration of population immunity in the most susceptible infant cohort. Conversely, the significant decline in PIV detection post-emergency warrants further investigation, as PIVs, particularly PIV 3, were primarily detected in the summer during the COVID-19 emergency phase. These distinct shifts in prevalence and age-specific burden necessitate proactive adjustments in clinical vigilance and prevention strategies, including vaccine timing and prioritization, to manage the altered infectious disease landscape.

In addition to single-virus prevalence, our data revealed that co-infections were common, with HRV/HEV and RSV being the most frequent dual detection (17.0% of co-infections). Notably, despite the resurgence of viruses and common co-infections, the average LOS was shorter in the post-emergency phase than in the COVID-19 emergency phase (3.2 vs. 4.3 days; *p* < 0.001). This decrease may be attributable to multiple factors, including the older average age of hospitalized children (who generally experience less severe disease), improved institutional readiness, faster diagnostic turnaround times (e.g., FilmArray^®^ utilization), and clinical experience gained by healthcare providers during the COVID-19 emergency. However, because standardized severity indicators (e.g., oxygen requirement metrics, ICU admission, and validated severity scores) were not consistently available across the full retrospective study period, we were unable to robustly quantify the clinical impact of co-infections in this study. The association between co-infections and outcomes such as disease severity or prolonged hospitalization remains an important priority for future studies integrating comprehensive clinical severity endpoints [28].

Looking ahead, the epidemiology of respiratory viruses is unlikely to return to the stable pre-pandemic baseline. Instead, circulation patterns will continue to evolve under the following influences: (1) viral evolution and mutation (such as the emergence of new IFV or RSV variants), (2) introduction of novel pathogens with pandemic potential, (3) possible re-emergence of pandemics due to global interconnectedness, and (4) development and deployment of vaccines. Indeed, recent progress in RSV immunization is expected to pose a significant challenge to previous viral epidemiology [9]. Not all respiratory viruses were affected uniformly by the COVID-19 period; rather, the observed changes likely reflect a combination of virus-specific properties (e.g., environmental stability/structure), dominant transmission routes, and population-level susceptibility shaped by prior exposure and immune dynamics [5,6,7,8]. Therefore, sustained surveillance of respiratory viruses is essential, not only for monitoring immediate clinical burden [21,29] but also for anticipating long-term shifts in viral ecology [7]. Tracking these patterns will help predict emerging threats, guide vaccine policy, and strengthen preparedness against future pandemics.

This study has several limitations. Due to its retrospective, single-center design in Yongin, South Korea, our findings may be influenced by local referral patterns and healthcare-seeking behaviors, limiting their generalizability to the national population. And the use of different testing platforms (Allplex vs. FilmArray, Table S1) introduced platform-dependent differences in target coverage. Specifically, this methodology biased pathogen detection, particularly for bocavirus, and limited our ability to consistently differentiate certain subtypes (e.g., HRV/HEV), requiring cautious interpretation of these trends. Because SARS-CoV-2 testing was frequently performed at external screening clinics and was not linkable to our dataset, in-hospital SARS-CoV-2 infections and co-detections are likely underestimated. Consequently, we cannot exclude the possibility that unrecognized concurrent or prior SARS-CoV-2 infections may influence clinical presentations. While we observed significant epidemiological shifts, we did not directly measure contextual factors such as school-related mixing, precise masking adherence, or other behavioral changes. Therefore, the extent to which these specific behaviors contributed to the heterogeneous shifts in seasonality and age distribution remains a hypothesis requiring further prospective or multicenter validation.

In conclusion, COVID-19 preventive measures effectively limited the spread of various respiratory viruses. While many respiratory viruses exhibited unpredictable seasonal patterns during the COVID-19 emergency phase, they resumed their typical seasonal patterns afterward. Analysis of data collected from hospitalized children with acute respiratory infections during and after the COVID-19 emergency revealed seasonal and age-related epidemiological changes between the two periods. This study could provide epidemiologic evidence that can inform clinical preparedness, diagnostic stewardship, and resource planning for pediatric respiratory virus epidemics in the post-emergency phase.

## Figures and Tables

**Table 1:** Comparison of viral targets between the two multiplex RT-PCR panels.

	Multiplex Real-Time PCR	FilmArray^®^
Reporting time	1 day	2–3 h
Off-hours test reporting	Not available	Available
Detection of pathogens other than viruses	Not included	Included
HRV and HEV differentiation	Available	Not available
RSV subtype differentiation	Available	Not available
Bocavirus detection	Available	Not available
Old CoV subtypes	3 available	4 available

Abbreviations: PCR, polymerase chain reaction; HRV, human rhinovirus; HEV, human enterovirus; RSV, respiratory syncytial virus; CoV, coronavirus.

**Table 2:** Demographic characteristics of children during and after the COVID-19 emergency phase.

Total	During the Emergency (2020–2022)	Post-Emergency (2023–2024)	*p*-Value
No. of patients	1724	1934	
Male, *n* (%)	955 (55.4)	1062 (54.9)	0.795
Age (years)	3.5 ± 4.1	4.9 ± 5.0	<0.001 ***
LOS (days)	4.3 ± 2.7	3.2 ± 2.6	<0.001 ***
Respiratory viruses detected, ***n*** (%)			
HRV/HEV	637 (36.9)	598 (30.9)	0.001 **
RSV A/B	261 (15.1)	264 (13.7)	0.217
PIV 1–4	265 (15.4)	218 (11.3)	<0.001 ***
ADV	99 (5.7)	242 (12.5)	<0.001 ***
MPV	87 (5.0)	87 (4.5)	0.484
IFV A/B	94 (5.5)	545 (28.2)	<0.001 ***

Data are presented as the number of patients (%) or the mean value ± standard deviation. ** *p* < 0.01, *** *p* < 0.001. Abbreviations: LOS, length of stay; HRV, human rhinovirus; HEV, human enterovirus; RSV, respiratory syncytial virus; PIV, parainfluenza virus; ADV, adenovirus; MPV, metapneumovirus; IFV, influenza virus.

**Table 3:** Comparison of respiratory viruses detected by age group during and after the COVID-19 emergency phase.

During the Emergency (2020–2022)		Respiratory Viruses Detected, *n* (%)					
Age Group (Years), *n* (%)		HRV/HEV	RSV A/B	PIV	ADV	MPV	IFV A/B
Infant (0–1)	270	103 (38.1)	58 (21.5)	44 (16.3)	9 (3.3)	8 (3.0)	5 (1.9)
Toddler (1–4)	956	436 (45.6)	184 (19.2)	183 (19.1)	77 (8.1)	52 (5.4)	6 (0.6)
Pre-schooler (4–8)	251	69 (27.5)	19 (7.6)	33 (13.1)	9 (3.6)	23 (9.2)	36 (14.3)
School-aged (8–13)	135	23 (17.0)	0 (0.0)	1 (0.7)	2 (1.5)	4 (3.0)	20 (14.8)
Adolescent (13–18)	112	6 (5.4)	0 (0.0)	3 (2.7)	2 (1.8)	0 (0.0)	27 (24.1)
**After the Emergency (2023–2024)**		**Respiratory Viruses Detected, *n* (%)**					
**Age Group (Years), *n* (%)**		**HRV/HEV**	**RSV A/B**	**PIV**	**ADV**	**MPV**	**IFV A/B**
Infants (0–1)	357	97 (27.2)	94 (26.3)	48 (13.4)	24 (6.7)	12 (3.4)	22 (6.2)
Toddler (1–4)	670	293 (43.7)	139 (20.7)	111 (16.6)	122 (18.2)	49 (7.3)	61 (7.9)
Pre-schooler (4–8)	432	140 (32.4)	26 (6.0)	36 (8.3)	80 (18.5)	20 (4.6)	169 (39.1)
School-aged (8–13)	244	58 (23.8)	4 (1.6)	11 (4.5)	15 (6.1)	3 (1.2)	133 (54.5)
Adolescent (13–18)	231	10 (4.3)	1 (0.4)	7 (3.0)	1 (0.4)	3 (1.3)	160 (69.3)

Abbreviations: HRV, human rhinovirus; HEV, human enterovirus; RSV, respiratory syncytial virus; PIV, parainfluenza virus; ADV, adenovirus; MPV, metapneumovirus, IFV; influenza virus.

## Data Availability

The data from this study are not available for sharing due to Institutional Review Board (IRB) regulations.

## Supplementary Materials

The following supporting information can be downloaded at: https://www.mdpi.com/article/10.3390/v18010130/s1, Table S1: Pathogen targets and reporting granularity by respiratory panel platform.

## Abbreviations

The following abbreviations are used in this manuscript:

ARI	acute respiratory infection
HRV	human rhinovirus
RSV	respiratory syncytial virus
PIV	parainfluenza virus
MPV	metapneumovirus
ADV	adenovirus
IFV	influenza virus
CoV	coronavirus
NPI	non-pharmaceutical intervention
SARS-CoV2	severe acute respiratory syndrome coronavirus 2
