# Supplementary material for: Changing Trends of Respiratory Viruses in Hospitalized Children During and After the COVID-19 Emergency Phase in Yongin, South Korea (2020–22 vs. 2023–24)
Source: Viruses. 2026 Jan 20;18(1):130. doi: 10.3390/v18010130 (PMC12846629; doi:10.3390/v18010130)
Supplement: Supplementary file 1 [file viruses-18-00130-s001.zip › viruses-4102908-supplementary.pdf]

Supplementary Table S1.

Table S1. Pathogen targets and reporting granularity by respiratory panel platform.

Category	Target	Allplex™ RP 1/2/3 (multiplex RT-PCR)	FilmArray® RP/RP2.1	Notes
**Virus**	Influenza A (with subtyping)	Yes	Yes	Allplex provides FluA subtyping; FilmArray reports FluA and subtypes depending on panel.
	Influenza B	Yes	Yes	
	Respiratory Syncytial virus	Yes	Yes	Allplex differentiates RSV A and B; FilmArray reports RSV without A/B subtype.
	Parainfluenza virus 1–4	Yes	Yes	
	Adenovirus	Yes	Yes	
	Human metapneumovirus	Yes	Yes	
	Human rhinovirus	Yes	Reported as HRV/enterovirus	FilmArray does not differentiate HRV from enterovirus.
	Human enterovirus	Yes	Reported as HRV/enterovirus	
	Human bocavirus	Yes	No	Not included in FilmArray® panel used at our institution.
	Seasonal coronaviruses 229E, NL63, OC43	Yes	Yes	
	Seasonal coronavirus HKU1	No	Yes	Not included in the Allplex™ RP 1/2/3 menu.
	SARS-CoV-2	Not in RP 1/2/3	Yes	In our dataset, many suspected cases were tested externally; single-target RT-PCR also used.

**Bacteria**	Mycoplasma pneumoniae	No	Yes	Detected by FilmArray®; bacterial targets were excluded from the main virus-focused analyses.
	Bordetella pertussis	No	Yes	
	Bordetella parapertussis	No	Yes	
	Chlamydia pneumoniae	No	Yes	
